# Complexes With Biologically Active Ligands. Part 10^1^ Inhibition of Carbonic Anhydrase Isozymes I and II With Metal Complexes of Imidazo[2,1−*b* ]-1,3,4-Thiadiazole-2-Sulfonamide

**DOI:** 10.1155/MBD.1997.19

**Published:** 1997

**Authors:** Andrea Scozzafava, Claudiu T. Supuran

**Affiliations:** Università degli Studi Dipartimento di Chimica Laboratorio di Chimica Inorganica e Bioinorganica Via Gino Capponi 7 Florence I-50121 Italy

## Abstract

The title compound was prepared by an improved variant of the literature procedure, and metal complexes containing its anion and the following metal ions: Zn(II), Cd(II), Hg(II), Co(II), Ni(II), Cu(II), V(IV), Fe(III) and Ag(I) were synthesized and characterized by standard procedures (elemental analysis; IR, electronic, NMR and EPR spectroscopy; TG, magnetic and conductimetric measurements). The parent sulfonamide and its metal complexes are potent inhibitors of two carbonic anhydrase (CA) isozymes, CA I and II, and they might possess applications as selective cerebrovasodilating agents.


### COMPLEXES WITH BIOLOGICALLY ACTIVE LIGANDS. Part 10[1] INHIBITION OF CARBONIC ANHYDRASE ISOZYMES I AND II WITH METAL COMPLEXES OF IMIDAZO[2,1-*b*]-1,3,4-THIADIAZOLE-2-SULFONAMIDE

Andrea Scozzafava and Claudiu T. Supuran*

Università degli Studi, Dipartimento di Chimica, Laboratorio di Chimica Inorganica e Bioinorganica, Via Gino Capponi 7, I-50121, Florence, Italy

**Abstract**: The title compound was prepared by an improved variant of the literature procedure, and metal complexes containing its anion and the following metal ions: Zn(II), Cd(II), Hg(II), Co(II), Ni(II), Cu(II), V(IV), Fe(III) and Ag(I) were synthesized and characterized by standard procedures (elemental analysis; IR, electronic, NMR and EPR spectroscopy; TG, magnetic and conductimetric measurements). The parent sulfonamide and its metal complexes are potent inhibitors of two carbonic anhydrase (CA) isozymes, CA I and II, and they might possess applications as selective cerebrovasodilating agents.

### Introduction

Heterocyclic sulfonamides possessing carbonic anhydrase (CA, EC 4.2.1.1) inhibitory properties are clinically used pharmacological agents in the treatment of a variety of diseases.[2] Thus, acetazolamide **1** and some other thiadiazole-sulfonamides derived from **2**, methazolamide **3**, ethoxzolamide **4** and sezolamide **5** represent several generation of such drugs, used in the last 40 years in the treatment or prevention of glaucoma,[2a,c,d] gastro-duodenal ulcers,[2b] mountain sickness[3] and other conditions associated with acid-base disequilibria.[2a]

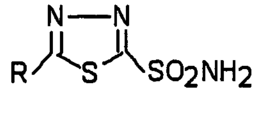

**1**: R = AcNH
**2**: R = $NH_2$

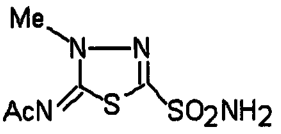

**3**

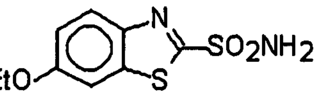

**4**

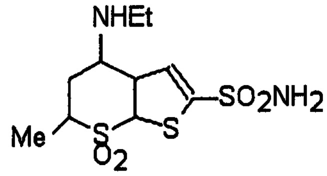

**5**

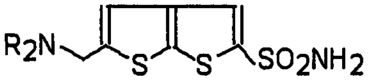

**6**: R = Alkyl; hydroxyalkyl

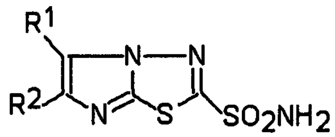

**7**: $R^1$= H, Me, Ph; $R^2$ = alkyl

**8**: $R^1$ = $R^2$ = H

As it was observed that sulfonamides possessing a bicyclic ring system such as **4** or **5** are generally stronger inhibitors for several of the eight CA isozymes presently known in vertebrates,[4] as compared to derivatives containing only one such homo- or heterocyclic ring system,[2a,5] much synthetic work has been devoted to the preparation and evaluation of such compounds, in order to obtain stronger and more selective inhibitors, for diverse medical applications.[6-8]

Compounds such as **6**[8a] and **7**, **8**[6a] not only showed excellent inhibitory properties against the major red cell isozyme, CA II,[2,3] but also possessed interesting pharmacological qualities, making them candidates for the development of topical antiglaucoma agents in the case of **6**,[7,8] and selective cerebrovasodilators, in the case of **7**.[6] As no selective such pharmacological agents are known up to now, the development of a selective cerebrovasodilator would constitute a good approach for the treatment of cerebrovascular disease, a condition becoming more stringent in the last period, as the number of elderly people is increasing.[6]

Recently, it was also reported[9,10] that metal complexes of heterocyclic sulfonamides such as **1-5** behave as even stronger CA inhibitors as compared to the ligands from which they derive. Thus, a large number of such derivatives were prepared and assayed as inhibitors of three CA isozymes up to now, i.e., CA I, II and IV, in the search for isozyme-specific inhibitors. Continuing our research for specific inhibitors, in this paper we report the preparation of metal complexes of sulfonamide **8** possessing very good CA inhibitory properties. The Zn(II), Cd(II), Hg(II), Co(II), Ni(II), Cu(II), V(IV), Fe(III) and Ag(I) complexes containing the conjugate base of this sulfonamide were obtained and characterized by standard procedures (elemental analysis, IR, electronic, NMR and EPR spectroscopy; TG, magnetic and conductimetric measurements).

**Materials and Methods**

IR spectra of KBr pellets were recorded on a Perkin-Elmer 16PC FTIR instrument, in the range 200-4000 $cm^{-1}$. Solution electronic spectra were recorded with a Cary 3 spectrophotometer interfaced with a PC. Electronic spectra were obtained by the diffuse reflectance technique in MgO as reference, with a Perkin Elmer Lambda 15 apparatus, in the range 200-900 $cm^{-1}$. Conductimetric measurements were done in DMF solutions, at 25°C (concentrations of 1 mM of complex) with a Fisher conductimeter. $^1$H-NMR spectra were recorded with a Bruker CPX-200 instrument working at 200 MHz, in DMSO-$d_6$ as solvent. Chemical shifts are expressed as $\delta$ values relative to $Me_4Si$ as external standard. EPR spectra were recorded on a Varian E-9 spectrometer at room temperature, in crystalline powder. The field was calibrated using crystalline diphenylpicrylhydrazyl (g = 2.0036). Magnetic susceptibility measurements were carried out at room temperature with a fully automated AZTEC DSM8 pendulum-type susceptometer. Mercury(II) tetrakis(thiocyanato)cobaltate(II) was used as a susceptibility standard. Corrections for the diamagnetism were estimated from Pascal's constants.[11] Elemental analyses were done by combustion for C,H,N with an automated Carlo Erba analyzer, and gravimetrically for the metal ions, and were 0.4% of the theoretical values. Thermogravimetric measurements were done in air, at a heating rate of 10°C/min., with a Perkin Elmer 3600 thermobalance.

Acetazolamide and methazolamide used in the enzymatic assay as standards and for the preparation of **2** and **8** were from Sigma. Sulfonamide **2** was prepared as described in the previous paper by deacetylation of acetazolamide,[1] whereas **8** was obtained by an improvement of the literature procedure[6a] by condensation of 5-amino-1,3,4-thiadiazole-2-sulfonamide **2** with diethyl chloroacetal (see later in the text). Diethyl chloroacetal was from Acros; metal salts and solvents were from Merck. Human CA I and CA II cDNAs were expressed in *Escherichia coli* strain BL21 (DE3) from the plasmids pACA/HCA I and pACA/HCA II (the two plasmids were a gift from Prof. Sven Lindskog, Umea University, Sweden). Cell growth conditions were those described by Lindskog's group,[12] and enzymes were purified by affinity chromatography according to the method of Khalifah et al.[13] Enzyme concentrations were determined spectrophotometrically at 280 nm, using a molar absorptivity of 49 $mM^{-1}$. $cm^{-1}$ for CA I and 54 $mM^{-1}.cm^{-1}$ for CA II, respectively, based on $M_r$ = 28.85 kDa for CA I, and 29.3 kDa for CA II, respectively.[14]

Initial rates of 4-nitrophenyl acetate hydrolysis were monitored spectrophotometrically, at 400 nm and 25°C, with a Cary 3 apparatus interfaced with an IBM compatible PC by the method of Pocker and Stone.[15]. Solutions of substrate were prepared in anhydrous acetonitrile; the substrate concentrations varied between $10^{-2}$ and $10^{-5}$ M. A molar absorption coefficient ($\varepsilon$ = 18,400 $M^{-1}.cm^{-1}$ was used for the 4-nitrophenolate formed by hydrolysis, in the conditions of the experiments (pH 7.80), as reported by Pocker and Stone.[15] Non-enzymatic hydrolysis rates were always subtracted from the observed rates. Duplicate experiments were done for each inhibitor, and the values reported throughout the paper are the averages of such results. $IC_{50}$ represents the molarity of inhibitor producing a 50% decrease of enzyme catalyzed hydrolysis of 4-nitrophenyl acetate.

**Synthesis of imidazo[2,1-*b*]-1,3,4-thiadiazole-2-sulfonamide 8**

1.80 g (10 mmol) of 5-amino-1,3,4-thiadiazole-2-sulfonamide **2** and 1.4 mL (10 mmol) of triethylamine were suspended in 50 mL of anhydrous acetonitrile, then 1.27 mL (11 mmol) of diethyl chloroacetal were added and the mixture was magnetically stirred at room temperature for 5 hours and then heated at refluxation for 24 hours. The title compound precipitated by cooling, was filtered and recrystallized from ethanol (69 % yield ). Presumably, a Schiff base intermediate is formed during the first step, as reported for related sulfonamide derivatives,[16] which subsequently cyclizes intramolecularly, in the presence of triethylamine, with formation of the imidazo-thiadiazole ring system.[6a] The title compound was previously reported as hydrobromide salt (m.p. 207-210°C)[6a] being obtained with a 26 % yield, by a synthetic procedure similar to the one described above, except for the lack of triethylamine in the reaction medium.[6a] As seen from our data, the use of the base leads to a highly improved synthesis yield. The title compound was obtained as a white powder, m.p. 255-258 °C; IR (KBr), $cm^{-1}$: 633, 709, 752, 915, 1030, 1175, 1358, 1540, 1610, 3160; UV (MeOH): $\lambda_{max}$ 262 nm (lg $\varepsilon$ = 2.75); $^1$H-NMR (DMSO-$d_6$), $\delta$, ppm: 7.45-7.92 (m, 2H, CH=CH); 8.48 (br s, 2H, $SO_2NH_2$). The last signal disappears after 5 min by addition of $D_2O$ into the NMR tube. Analysis, found: C, 23.3; H, 2.0; N 27.0 %; $C_4H_4N_4O_2S_2$ requires: C, 23.5; H, 1.9; N, 27.4%.

**General procedure for the preparation of metal complexes 9-17**

An amount of 0.102 g (0.5 mmol) of sulfonamide **8** were suspended in 10 mL MeOH and the calculated amount of aqueous 1 N NaOH solution was added in order to obtain the sodium salt $RSO_2NHNa$. This was treated thereafter with an aqueous solution of the metal salt ($AgNO_3$; chlorides for Zn(II), Cd(II), Hg(II), Cu(II), Co(II) and Ni(II); vanadyl sulfate, and Fe(III) perchlorate), working at molar ratios $M^{n+}$ : $RSO_2NH^-$ of 1:1 (for the Ag(I) derivatives); 1:2 (for the divalent metal ions and V(IV)); and 1:3 (for the Fe(III) derivative), respectively. The reaction mixture was heated on a steam bath for 3 hours, then the precipitated complexes were filtered, thoroughly washed with cold water and alcohol. Crystallization was not done as the only solvents in which the complexes have good solubility are DMSO and DMF. The complexes **9-17** melt with decomposition at temperatures higher than 330 °C.

**Results and Discussion**

Barnish et al.[6a] reported the synthesis and murine erythrocyte CA (a mixture of isozymes CA I and CA II)[2] inhibitory properties of a series of imidazo-1,3,4-thiadiazole-sulfonamides of type **7**, prepared in the search of a selective cerebrovasodilating agent. As the parent compound of this homologous series, **8**, possesses an interesting donor system for obtaining metal complexes, we reinvestigated its synthesis and properties. The general synthetic method for obtaining this ring system involves condensation of 5-amino-1,3,4-thiadiazole-2-sulfonamide **2** with (α-halogeno-ketones (or halogenoacetaldehyde or their functional derivatives in the case of the parent compound **8**). The synthetic procedures used by us is shown in Scheme 1.

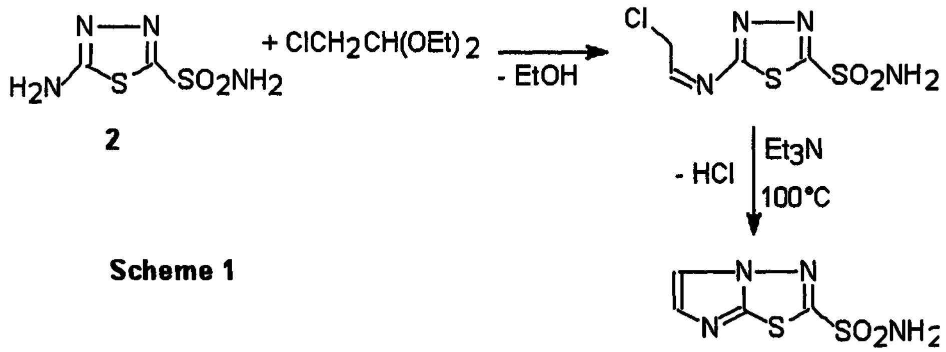

**Scheme 1**

Thus, condensation of **2** with diethyl chloroacetal in acetonitrile, at room temperature initially, and then at reflux, in the presence of triethylamine, led to imidazo[2,1-*b*]-1,3,4-thiadiazole-2-sulfonamide **8** with a 69% yield. The highly increased yield in the presence of triethylamine prompted us to hypothesize that the reaction proceeds in two steps, as shown in Scheme 1. In the first one, occurring at room temperature, a Schiff base is probably formed, which was not isolated, as it reacted in the second step to form the desired product. This type of reaction involving Schiff bases has in fact been thoroughly investigated by this group[16] for the reaction of **2** as well as other amino-sulfonamides with a large series of aldehydes and ketones. In the second step, taking place at higher temperatures, the Schiff base cyclizes intramolecularly with formation of hydrochloric acid and the imidazo-thiadiazole ring system. The presence of triethylamine presumably favors this step, and might explain the improved yield of our synthetic variant, as compared to the original one of Barnish et al.[6a] The obtained compound was extensively characterized by physico-chemical methods (see Materials and Methods) which confirmed the proposed structure (only melting point and elemental analysis have been reported for **8** in ref.[6a]).

The metal complexes containing the conjugate base of sulfonamide **8** and transition metal ions are shown in Table I, together with their elemental analysis data (0.4% of the theoretical values).

The newly prepared compounds, **9-17**, have also been characterized by spectroscopic (IR, EPR, electronic, and $^1H$-NMR), magnetic, conductimetric and thermogravimetric (TG) measurements. Some of these data are shown in Tables II and III.

In the IR spectra of complexes **9-17**, the following features were observed: (i) the shift of the two sulfonamido vibrations to lower wavenumbers (with 5-15 $cm^{-1}$ for the symmetric vibration, and 8-18 $cm^{-1}$ for the antisymmetric one, respectively), as compared to the corresponding bands in the spectra of the original sulfonamide **8**; (ii) a similar shift of the C=N vibration (at 1610 $cm^{-1}$ in **8**), generally atributed to the thiadiazolic moiety,[9,10,16] which for the complexes appears at 1600 $cm^{-1}$. The only exception is constituted by the silver complex **17**, for which two broad bands are seen in this region, one at 1580 $cm^{-1}$ and the other one at 1610 $cm^{-1}$, but higly reduced in intensity as compared to the corresponding band of **8**; (iii) the absence of ν(NH) vibrations, which for **8** appeared at 3160 $cm^{-1}$; (iv) the presence of ν(OH) bands, at around 3400 $cm^{-1}$ in the spectra of complexes **14** and **15** containing coordinated water molecules (data not shown). All these data suggest the participation of the deprotonated sulfonamido moiety and endocyclic nitrogen(s) of the ligand in coordinating the metal ions in the prepared complexes.

Table I: Prepared complexes **9-17**, containing the conjugate base of sulfonamide **8** and their elemental analysis data (L stands for the sulfonamide deprotonated species of **8**).

| No. | Complex | Color | Yield (%) | Analysis (calculated/found) %M[a] | %C[b] | %H[b] | %N[b] |
|---|---|---|---|---|---|---|---|
| **9** | $[ZnL_2]$ | white | 81 | 13.8/13.5 | 20.3/20.1 | 1.2/1.0 | 23.7/23.4 |
| **10** | $[CdL_2]$ | white | 87 | 21.7/21.3 | 18.5/18.6 | 1.1/0.8 | 21.6/21.5 |
| **11** | $[HgL_2]$ | white | 93 | 33.0/33.2 | 15.8/15.7 | 0.9/0.9 | 18.4/18.0 |
| **12** | $[VOL_2]$ | gray | 79 | 10.7/11.1 | 20.3/20.1 | 1.2/1.3 | 23.6/23.3 |
| **13** | $[FeL_3]$ | brown | 94 | 8.4/8.0 | 21.6/21.5 | 1.3/1.3 | 25.2/25.0 |
| **14** | $[CoL_2(OH_2)_2]$ | pink | 62 | 11.7/11.7 | 19.1/18.8 | 2.0/2.1 | 22.3/22.1 |
| **15** | $[NiL_2(OH_2)_2]$ | green | 84 | 11.7/11.8 | 19.1/19.2 | 1.9/1.6 | 22.3/21.9 |
| **16** | $[CuL_2]$ | blue | 73 | 13.5/13.7 | 20.4/20.2 | 1.2/0.9 | 23.8/23.6 |
| **17** | $[AgL]_n$ | white | 95 | 34.6/34.7 | 15.4/15.7 | 0.9/0.9 | 18.0/17.9 |

[a]By gravimetry; [b]By combustion.

Table II: Spectroscopic, thermogravimetric and conductimetric data for compounds **8-17**.

| **Comp.** | **IR Spectra**[a], $cm^{-1}$ $(SO_2)^s$ ; $(SO_2)^{as}$ | (C=N) | **Electronic Spectra**[b] λ(nm) (lgε) | **TG analysis**[c] calc./found | **Conductimetry**[d] $\Lambda_M$ ($ohm^{-1}$ x $cm^2$x $mol^{-1}$) |
|---|---|---|---|---|---|
| **8** | 1175; 1358 | 1610 | 262 (2.75) | e | 11 |
| **9** | 1170; 1346 | 1600 | 271 (3.05) | e | 7 |
| **10** | 1170; 1347 | 1600 | 278 (3.12) | e | 8 |
| **11** | 1170; 1345 | 1595 | 276 (3.09) | e | 10 |
| **12** | 1163; 1340 | 1600 | 270 (3.06) | e | 11 |
| **13** | 1165; 1344 | 1600 | 272 (3.21) | e | 9 |
| **14** | 1170; 1345 | 1600 | 277 (3.08) | 7.1/6.9[f] | 11 |
| **15** | 1170; 1345 | 1600 | 277 (3.01) | 7.1/7.1[f] | 10 |
| **16** | 1170; 1340 | 1600 | 275 (3.11) | e | 9 |
| **17** | 1160; 1350 | 1580; 1610 | 289 (4.09) | e | 6 |

[a] In KBr; [b]In DMSO; [c]Weight loss between 130-200 °C; [d] $10^{-3}$ M solution, in DMF, at 25°C; [e] No weight loss seen under 250 °C; [f] Corresponding to two coordinated water molecules, lost at 180-190 °C.

Table III: Diffuse reflectance spectra, magnetic moments and proposed geometries for complexes **9-17**.

| **Complex** | **Electronic spectra** (λ, $cm^{-1}$)[a] | $\mu_{eff}$ (BM)[b] | **Geometry** |
|---|---|---|---|
| **9** | c | d | tetrahedral |
| **10** | c | d | tetrahedral |
| **11** | c | d | tetrahedral |
| **12** | 25,760; 15,400; 11,800(sh) | 1.85 | square pyramidal |
| **13** | 24,600; 20,500; 10,500 | 5.74 | octahedral |
| **14** | 25,200; 20,700(sh); 15,300 | 4.90 | octahedral |
| **15** | 17,600; 11,500 | 3.40 | octahedral |
| **16** | 14,500 | 1.98 | distorted tetrahedral |
| **17** | c | d | lineal |

[a] In MgO as standard material; [b] At room temperature; [c] No transitions seen; [d] Diamagnetic.

Solution electronic spectra of the complexes and the sulfonamide **8** confirmed the above mentioned assumption. Thus, the characteristic band of the conjugated thiadiazolo-sulfonamide system,[10,16] appearing at 262 nm in the spectrum of **8**, undergoes bathochromic shifts (with 8-17 nm) and hyperchromic effects in the sodium salt of **8** (data not shown) as well as the complexes **9-17**, similarly to other metal complexes of sulfonamides of type **1-5** previously reported by us.[9,10]

Conductimetry proved the non-electrolyte nature of **8** and of all complexes **9-17**, whereas TG analysis detected the presence of two coordination water molecules in the Co(II) and Ni(II) derivatives (Table II). No decomposition steps under 250°C were evidenced for the other metal complexes, whereas at higher temperatures intricate decomposition patterns were observed, with oxidation of the organic moieties of the complexes (data not shown).

Diffuse reflectance electronic spectra and magnetic susceptibility data at room temperature of the complexes containing paramagnetic metal ions (Table III) indicated the geometry of the central metal ions, which are square pyramidal for V(IV),[17] octahedral for Fe(III),[18] Co(II),[19] and Ni(II),[20] and presumably distorted terahedral for Cu(II).[21] All these derivatives present electronic spectra and magnetic moments characteristic of these metal ions in the above mentioned surroundings.[17-21] In the EPR spectrum of the Cu(II) complex **16**, a large signal was detected with $g_{\perp}$ = 1.95 and $g_{//}$ = 2.23, characteristic of Cu(II) in distorted tetrahedral geometry.[21]

The diamagnetic complexes **9-11** and **17** had $^1$H-NMR spectra similar to that of **8**, except for the fact that the $SO_2NH_2$ resonance, a broad singlet (2H) at 8.48 ppm in the spectrum of **8**, appeared as very broad signals at 8.50-8.55 ppm in the spectra of the complexes (data not shown), which were integrated for 1H, obviously due to the fact that the sulfonamido moiety was deprotonated.

The above data prompted us to propose tetrahedral geometries for Zn(II), Cd(II) and Hg(II) in the newly prepared compounds, whereas the silver derivative probably contains Ag(I) in a linear geometry.

Thus, the donor system of the bidentate ligand used for the preparation of metal complexes reported here is quite similar to those of acetazolamide **1**, methazolamide **3** and ethoxzolamide **4**,[9,10] consisting of the deprotonated sulfonamide moiety and the endocyclic N-3 atom of the thiadiazolic ring. An exception seems to be the Ag(I) derivative **17**, possessing IR and electronic spectra quite different of the other prepared complexes, which prompted us to hypothesize that the ligand acts monodentately in this case, by means of the deprotonated sulfonamido moiety, whereas the second coordination position of Ag(I) is occupied by the imidazolic nitrogen of another ligand molecule, leading thus to a polynuclear derivative as shown schematically below. In fact, some Ag(I) complexes of aromatic (bactericidal) sulfonamides were shown to possess such a structure.[22] The proposed structures for the new derivatives are shown below.

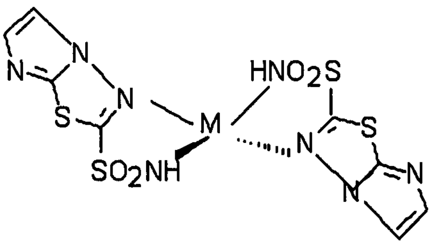

**9**: M = Zn; **10**: M = Cd

**11**: M = Hg; **16**: M = Cu

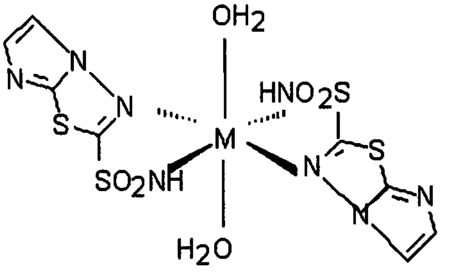

**14**: M = Co; **15**: M = Ni

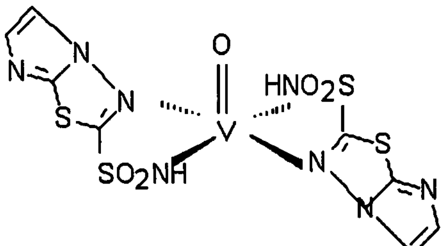

**12**

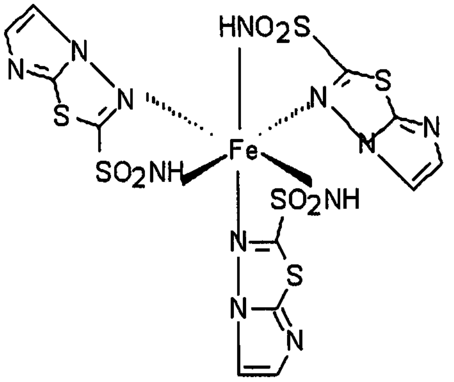

**13**

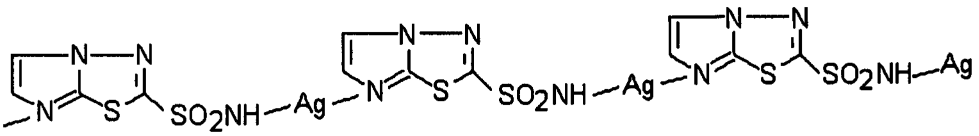

**17**

The presence of the imidazolic nitrogen atom of the ligand in complexes **9-16**, which presumably participates in coordination of the Ag(I) ions in complex **17**, but is free in the other compounds, prompted us to try the preparation of polynuclear complexes involving this particular atom. Thus, treatment of a solution of the Zn(II) derivative **9** in DMSO with an equimolar amount of $CuCl_2.2H_2O$ dissolved in the same solvent led to a clear greenish-blue solution, from which, by addition of diethyl ether precipitated a green powder (complex **18**) which by analysis led to the following minimal formula: $[CuZnL_2Cl_2]$ (found: Cu, 12.1; Zn, 12.0; C, 17.6; H, 0.8; N, 20.5; Cl, 13.0%; $CuZnC_8H_6N_8O_4S_4Cl_2$ requires: Cu, 11.8; Zn, 12.2; C, 17.9; H, 1.1; N, 20.9; Cl, 13.2%). The complex is a non-electrolyte, having an IR and solution electronic spectra highly similar to those of complex **16**. Importantly, in the IR spectrum of **18**, similarly to that of the Ag(I) derivative **17**, the C=N vibration is splitted: a band at 1610 and another one at 1580 $cm^{-1}$ were evidenced (data not shown). These data prompted us to propose the structure below for this polynuclear complex.

Compounds **8-18** prepared in this work were assayed as inhibitors of two pure CA isozymes (**8** has been tested previously[6a] against crude red cell murine CA, which is a mixture of CA I and CA II,[2] behaving as a very potent inhibitor, with a $K_I$ of 0.65 nM, determined by the method of Philpot and Philpot, which is a pH-changing method monitoring the hydration of $CO_2$ in the presence of the enzyme and its inhibitors). Our inhibition data, determined by the method of Poker and Stone,[15] for another reaction catalyzed by these enzyme, i.e., ester hydrolysis, are shown in Table IV.

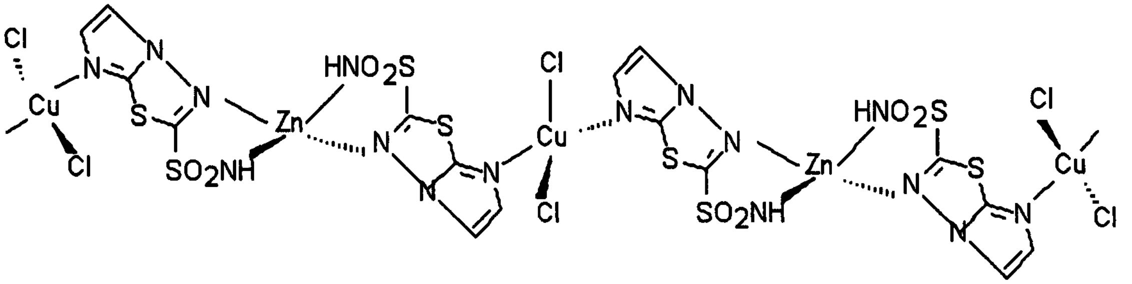

**18**

Table IV: Biological activity data of sulfonamide CA inhibitors and their metal complexes ($IC_{50}$ - the mean of two different assays - represents the molarity of inhibitor producing a 50% decrease of enzyme specific activity for the *p*-nitrophenyl acetate hydrolysis reaction)[15].

| Compound | $IC_{50}$ (μM) CA I[a] | CA II[a] |
|---|---|---|
| **1 (acetazolamide)** | 90 | 1.10 |
| **3 (methazolamide)** | 120 | 3.50 |
| **8** | 20.8 | 0.15 |
| **9** | 10.5 | 0.09 |
| **10** | 8.4 | 0.05 |
| **11** | 8.0 | 0.03 |
| **12** | 14.7 | 0.10 |
| **13** | 40.2 | 0.55 |
| **14** | 5.8 | 0.03 |
| **15** | 7.0 | 0.05 |
| **16** | 2.5 | 0.03 |
| **17** | 2.0 | 0.03 |
| **18** | 1.8 | 0.02 |

[a]Human (cloned) isozyme;

As seen from data of Table IV, all the prepared complexes act as very effective inhibitors against both CA isozymes, being in fact much better than the clinically used and potent inhibitors acetazolamide 1 and methazolamide 3 (used as standards). Our inhibition data confirmed previous data of Barnish et al.[6a] that compound **8** is a very potent inhibitor (although the assay methods and the enzyme preparations in the two studies are different, which explains in fact the diverse inhibition parameters obtained). As for other metal complexes of sulfonamide CA inhibitors, previously reported by us,[9,10] the newly prepared complexes act generally as much more potent inhibitors than the parent sulfonamide. In this case the only exception is the iron derivative **13** which, although being a stronger inhibitor than acetazolamide, is less effective than **8**, against both isozymes. One should note the good inhibitory properties of the new complexes against CA I (an isozyme more resistant to inhibition by sulfonamides, as compared to CA II), which is an exciting discovery due to the fact that this isozyme is highly abundant in vascular endothelium[24]
where it mediates vascular tonus and other physiological processes.[2c,6,24] The Ag(I) derivative **17** and the polynuclear compound **18** are particularly active in inhibiting this isozyme.

In conclusion, this study reports an efficient synthetic method for a strong CA inhibitor, imidazo-[2,1-*b*]-1,3,4-thiadiazole-2-sulfonamide, the preparation and characterization of some of its mono- and polynuclear metal complexes, and their inhibitory effect against two isozymes, CA I and CA II of human origin. The new complexes are effective inhibitors of both CAs, showing notable inhibition against CA I, an enzyme involved in vascular processes.

**Acknowledgments**: This research was financed in part by the EU grant ERB CIPDCT 940051. Thanks are addressed to Professor Sven Lindskog (Umea University, Sweden) for the gift of the plasmids expressing CA I and CAII.

**References**

1. Preceding part: A. Jitianu, M.A. Ilies, F. Briganti, A. Scozzafava, C.T. Supuran, *Metal Based Drugs.*, **1997**, *4*, 1-8.
2. a) C.T. Supuran, "Carbonic anhydrase inhibitors", in "Carbonic Anhydrase and Modulation of Physiologic and Pathologic Processes in the Organism", I. Puscas Ed., Helicon, Timisoara **1994**, pp. 29-111; b) I. Puscas, C.T. Supuran, "Farmacologia clinica da ulcera peptica" in "Aparelho Digestivo", J. Coelho Ed., MEDSI, Rio de Janeiro, **1996**, pp. 1704-1734; c) T.H. Maren, *Pharmacol.Rev.*, **1967**, *47*, 595-782; d) T.H. Maren, *J. Glaucoma*, **1995**, *4*, 49-62.
3. E.B. Larson, R.C. Roach, R.B. Schoene, T.F. Hornbein, *JAMA*, **1982**, *248*, 328-332.
4. D. Hewett-Emmett, R.E. Tashian, *Mol.Phylogenet.Evol.*, **1996**, *5*, 50-77.
5. C.T. Supuran, *Roum.Chem.Quart.Rev.*, **1993**, *1*, 77-116.
6. a) I.T. Barnish, P.E. Cross, R.P. Dickinson, B. Gadsby, M.J. Parry, M.J. Randall, I.W. Sinclair, *J.Med. Chem.*, **1980**, *23*, 117-121; b) P.E. Cross, B. Gadsby, G.F. Holland, W.M. McLamore, *J.Med.Chem.*, **1978**, *21*, 845-851.
7. a) G.D. Hartmann, W. Halczenko, *Heterocycles*, **1989**, *29*, 1943-1949; b) O.W. Waltersdorf, H. Schwam, J.B. Bicking, S.J. Solms, D.R. Fishman, S.L. Graham, P. Gautheron, J.M. Hoffman, R.D. Larsson, W.S. Lee, S.R. Michelson, C.M. Robb, N.N. Share, K.L. Shepard, A.M. Smith, J.M. Sondey, K.M. Strohmaier, M.F. Sugrue, M.P. Viader, *J.Med.Chem.*, **1989**, *32*, 2486-2492; c) S.L. Graham, K.L. Shepard, P.S. Anderson, J.J. Baldwin, D.B. Best, M.E. Christy, M.B. Freedman, P. Gautheron, C.N. Habecker, J.M. Hoffman, P.A. Lyle, S.R. Michelson, G.S. Ponticello, C.M. Robb, H. Schwam, A.M. Smith, R.L. Smith, J.M. Sondey, K.M. Strohmaier, M.F. Sugrue, S.L. Varga, *J.Med.Chem.*, **1989**, *32*, 2548-2554; d) S.L. Graham, J.M. Hoffman, P. Gautheron, S.R. Michelson, T.H. Scholz, H. Schwam, K.L. Shepard, A.M. Smith, J.M. Sondey, M.F. Sugrue, R.L. Smith, *J.Med.Chem.*, **1990**, *33*, 794-799.
8. a) J.D. Prugh, G.D. Hartmann, P.J. Mallorga, B.M. McKeever, S.R. Michelson, M.A. Murcko, H. Schwam, R.L. Smith, J.M. Sondey, J.B. Springer, M.F. Sugrue, *J.Med.Chem.*, **1991**, *34*, 1805-1818; b) T.J. Blacklock, P. Sohar, J.W. Butcher, T. Lamanec, E.J.J. Grabowski, *J.Org.Chem.*, **1993**, *58*, 1672-1679; c) C. A. Hunt, P.J. Mallorga, S.R. Michelson, H. Schwam, J.M. Sondey, R.L. Smith, M.F. Sugrue, K.L. Shepard *J.Med.Chem.*, **1994**, *37*, 240-247.
9. a) G. Alzuet, S. Ferrer, J. Borras, C.T. Supuran, *Roum.Chem.Quart.Rev.*, **1994**, *2*, 283-300; b) C.T. Supuran, R. Stefan, G. Manole, I. Puscas, M. Andruh, *Rev.Roum.Chim.*, **1991**, *36*,1175-1179; c) C.T. Supuran, G. Manole, M. Andruh, *J.Inorg.Biochem.*, **1993**, *49*, 97-103; d) C.T. Supuran, *Rev.Roum.Chim.*, **1992**, *37*, 849-855; e) C.T. Supuran, M. Andruh, *Rev.Roum.Chim.*, **1994**, *39*, 1229-1234.
10. a) C.T. Supuran, *Rev.Roum.Chim.*, **1993**, *38*, 229-236; b) S.L. Sumalan, J. Casanova, G. Alzuet, J. Borras, A. Castiñeiras, C.T. Supuran, *J.Inorg.Biochem.*, **1996**, 62, 31-39; c) C.T. Supuran, G.L. Almajan, *Main Group Met. Chem.*, **1995**, *18*, 347-351; d) C.T. Supuran, *Metal Based Drugs*, **1995**, *2*, 327-330; e) C.T. Supuran, *Metal Based Drugs*, **1995**, *2*, 331-336; f) J. Borras, T. Cristea, C.T. Supuran, *Main Group Met. Chem.*, **1996**, *19*, 339-346; g) J. Borras, J. Casanova, T. Cristea, A. Gheorghe, A. Scozzafava, C.T. Supuran, V. Tudor, *Metal Based Drugs*, **1996**, *3*, 143-148.
11. R.S. Drago, in "Physical Methods in Chemistry", W.B. Saunders & Co., London, **1977**, p. 411.
12.a) C. Forsman, G. Behravan, A. Osterman, B.H. Jonsson, *Acta Chem. Scand.*, **1988**, *B42*, 314-318; b) G. Behravan, P. Jonasson, B.H. Jonsson, S. Lindskog, *Eur.J.Biochem.*, **1991**, *198*, 589-592.
13. R.G. Khalifah, D.J. Strader, S.H. Bryant, S.M. Gibson, *Biochemistry*, 1977, *16*, 2241-2247.
14. a) P.O. Nyman, S. Lindskog, *Biochim.Biophys.Acta*, **1964**, *85*, 141-151; b) L.E. Henderson, D. Henriksson, P.O. Nyman, *J.Biol.Chem.*, **1976**, *251*, 5457-5463.
15. Y. Pocker, J.T. Stone, *Biochemistry*, **1967**, *6*, 668-679.
16. a) C.T. Supuran, A. Nicolae, A. Popescu, *Eur.J. Med.Chem.*, **1996**, *31*, 431-438; b) C.T. Supuran, A. Popescu, M. Ilisiu, A. Costandache, M.D. Banciu, *Eur.J.Med.Chem.*, **1996**, *31*, 439-447; c) C.T. Supuran, A. Scozzafava, A. Popescu, R. Bobes-Tureac, A. Banciu, A. Creanga, G. Bobes-Tureac, M.D. Banciu, *Eur.J.Med.Chem.*, **1997**, *32*, (in press).
17. C.J. Balhausen, H. Gray, *Inorg.Chem.*, **1962**, *1*, 111-115.
18. A.B.P. Lever, "Inorganic Electronic Spectroscopy", 2nd Edition, Elsevier, Amsterdam, **1984**, pp. 135-138.
19. L. Banci, A. Bencini, C. Benelli, D. Gatteschi, C. Zanchini, *Struct.Bonding*, **1982**, *52*, 37-79.

20. L. Sacconi, F. Mani, A. Bencini, "Nickel", in "Comprehensive Coordination Chemistry", G. Wilkinson, R. Gillard and J. McCleverty Eds., Pergamon Press, Oxford, **1987**, Vol. 5, pp. 1-347.
21. a) D.W. Smith, *Struct.Bonding*, **1978**, *35*, 88-117; b) C.T. Supuran, C.I. Lepadatu, R. Olar, A. Meghea M. Brezeanu, *Rev.Roum.Chim.*, **1993**, *38*, 1509-1517; c) S. Ferrer, J.G. Haasnoot, R.A.G. de Graaf, J. Reedijk, J. Borras, *Inorg.Chim.Acta*, **1992**, *192*, 129-133; d) C.T. Supuran, *Metal Based Drugs*, **1996**, *3*, 79-83.
22. A. Bult, *Met. Ions Biol. Syst.*, **1983**, *16*, 261-268.
23. F.J. Philpot, J.L. Philpot, *Biochem. J.*, **1936**, *30*, 2191-3003.
24. a) M. Coltau, I. Puscas, C.T. Supuran, "Angiotensin II stimulates the activity of carbonic anhydrase I while administration of angiotensin converting enzyme inhibitors reduces the activity of isozyme CA I", in "Carbonic Anhydrase and Modulation of Physiologic and Pathologic Processes in the Organism", I. Puscas Ed., Helicon, Timisoara **1994**, pp. 273-275; b) I. Puscas, M. Coltau, I.C. Puscas, C.T. Supuran, *Rev.Roum.Chim.*, **1994**, *39*, 237-242.

**Received: January 14, 1997 - Accepted: January 30, 1997 - Received in revised camera-ready format: January 31, 1997**

